# Supplementary material for: Avoiding catastrophic overfitting in fast adversarial training with adaptive similarity step size
Source: PLoS One. 2025 Jan 7;20(1):e0317023. doi: 10.1371/journal.pone.0317023 (PMC11706396; doi:10.1371/journal.pone.0317023)
Supplement: Attachment — Submitted filename: Review.pdf [file pone.0317023.s001.pdf]

Title: Avoiding Catastrophic Overfitting in Fast Adversarial Training with Adaptive Similarity Step Size

The article discusses strengths and weaknesses of adversarial learning when training deep neural network models. A new method implemented by the authors, called “Fast adversarial training with adaptive similarity step size (ATSS)”, is presented and tested on ResNet18 and VGG19 models by using CIFAR10, CIFAR100 and Tiny ImageNet datasets. The results confirm the validity of the approach, since ATSS avoids catastrophic overfitting, without extra computational costs

General

The article is well written and organized, the quality of English is good. I found some minor issues, which I listed below. The description of the ATSS algorithm is well structured and allows the readers to fully understand how the method works. The presentation of results on benchmark datasets could be improved in order to enhance a full comprehension: did the authors use pre-trained weights for ResNet18 and VGG19? This information is relevant. Moreover, sometimes the authors do not fully explain their outcomes (see below). Finally, the ATSS method achieves better results than other techniques with respect to accuracy, but the advantage seems limited. I understand the difficulty of achieving significantly better performances, but the authors might add some plots/statistical data supporting their claims.
Overall, the authors need to substantially modify the presentation of the experiments and their results in order to improve the quality of the article and meet the Journal requirements.

Detailed

The formatting of the paper is not justified. I suggest the authors change it.

When describing data in Tables 1 and 2, the authors do not mention the last line of both tables, that is data concerning the case “FGSM-RS(α=10/255)”. Therefore, I guess that this condition is irrelevant. Why did they insert this information?

When presenting the experimental setup, the authors state “The optimizer used in the experiments is the SGD optimizer, with a momentum of 0.9 and a weight decay coefficient of 5e-4”. Did they consider the possibility of using the Adam optimizer [1]?

[1] Kingma, D. P. & Ba, J. (2014). Adam: A method for stochastic optimization. *arXiv preprint arXiv:1412.6980.*

When the authors discuss the results of the experiments about prevention of catastrophic overfitting, they claim “Throughout the 100 epochs of adversarial training, the ResNet18 and VGG19 models maintain a consistent level of classification accuracy against the multi-step attack method PGD, without losing robustness due to catastrophic overfitting”. What about FGSM? They do not say anything about this technique.

Errors

Section Introduction, page 1: in the sentence “Szegedy et al. discovered that minor input perturbations, nearly imperceptible to the human eye, can cause severe classification errors in DCNNs. [4]”, move the cited reference before the dot.

Section Introduction, page 2: in the sentence “Common adversarial training approaches, such as the Projected Gradient Descent (PGD) adversarial training proposed by Madry et al. [9] and the TRADES proposed by Zhang et al. [10], employ multi-step iterative processes to generate adversarial examples, which are then used to train the model”, the acronym TRADES is not explained. I recommend the authors to add this information.

Section Introduction, page 2: in the sentence “The experimental results demonstrate that our method successfully avoids catastrophic overfitting while achieving high robustness accuracy and clean accuracy..”, please remove one of the two dots.

Section Related Work (Adversarial examples and adversarial attack methods), page 3: in the sentence “Additionally, there are optimization-based adversarial attack methods, such as C&W attack proposed by Carlini et al. [19].”, I recommend the authors to provide the meaning of the C&W acronym.

Section Related Work (Adversarial examples and adversarial attack methods), page 4: in the sentence “AutoAttack combines multiple advanced attack methods, including APGD-CE [20], APGD-DLR [20], FAB [21], and Square Attack [22], with the goal of systematically evaluating the robustness of neural network models.”, can the authors provide the meanings of the acronyms APGD-CE, APGD-DLR and FAB?

Section Related Work (Adversarial defense methods), page 5: in the sentence “Similarly, Jia et al. proposed FGSM-MEP [28], which introduces a momentum mechanism that accumulates the adversarial perturbations generated in previous iterations and uses them as the initial perturbation for the next iteration, with periodic resets after a certain number of iterations”, I recommend the authors to add the explanation of the FGSM-MEP acronym.

Section ATSS, page 8: can the authors explain the symbol *f* in equation 7?

Section Experiments (Datasets), page 9: in the sentence “In the experimental section, we use three publicly available benchmark datasets: CIFAR-10, CIFAR-100, and Tiny ImageNet.”, can the authors insert references to both CIFAR-10 [2], CIFAR-100 [2] and Tiny ImageNet [3] datasets? I report the references here below:

[2] Krizhevsky, A., & Hinton, G. (2009). Learning multiple layers of features from tiny images.
[3] Le, Y., & Yang, X. (2015). Tiny imagenet visual recognition challenge. *CS 231N*, *7*(7), 3.

Section Experiments (Datasets), page 9: in the sentence “The Tiny ImageNet dataset is a smaller version of the ImageNet dataset, divided into 200 categories, containing 100,000 training images and 10,000 testing images”, can the authors insert references to the ImageNet [4] dataset? I report the references here below:

[4] Deng, J., Dong, W., Socher, R., Li, L. J., Li, K., & Fei-Fei, L. (2009). Imagenet: A large-scale hierarchical image database. In *2009 IEEE conference on computer vision and pattern recognition* (pp. 248-255). IEEE.

Section Experiments (Experimental setup), page 9: in the sentence “The influence coefficient β is set to 0.04, the standard step size $\alpha0$ is set to 10/255.”, the authors could add “(see section Hyperparameter Experiments)” at the end of the phrase, since they later explain how they choose these two values.

Section Experiments (Experimental setup), page 10: in the sentence “The CW adversarial attack method used is based on modifying the CW loss with PGD, with 20 iterations.”, the authors use the CW acronym, while they previously employed the C&W acronym. I recommend the authors to keep coherence in the text.

Section Experiments (Experiments on Adversarial Robustness and Training Cost), page 10: in the sentence “All methods are trained for 60 epochs, and the total training time is recorded. For robustness testing, we use five attack methods, i.e., FGSM, PGD-10, PGD-50, CW, and AA, to attack the trained models and record the classification accuracy under these attacks.”, the authors use the CW acronym, while they previously employed the C&W acronym. Still I suggest the authors use the same acronym across the whole manuscript.

Section Experiments (Experiments on Adversarial Robustness and Training Cost), page 11: in Table 4, can the authors modify the last column from “training time” to “Training time”?
Section Conclusion, page 13: in the sentence “In future’s work, more complex and sophisticated adaptive step size strategies could be explored to further enhance the model’s robustness.”, replace “future’s work” with “future works”.
